# Repercussions of the *Emergency neurological life support* on scientific literature: a bibliometric study

**DOI:** 10.1055/s-0043-1777110

**Published:** 2024-01-25

**Authors:** Miguel Bertelli Ramos, Matheus Machado Rech, João Paulo Mota Telles, Willian Medeiros Moraes, Manoel Jacobsen Teixeira, Eberval Gadelha Figueiredo

**Affiliations:** 1 Hospital do Servidor Público Estadual de São Paulo, Departamento de Neurocirurgia, São Paulo SP, Brazil.; 2 Universidade de Caxias do Sul, Faculdade de Medicina, Departamento de Neurocirurgia, Caxias do Sul RS, Brazil.; 3 Universidade de São Paulo, Faculdade de Medicina, Departamento de Neurologia, São Paulo SP, Brazil.; 4 Hospital Nossa Senhora da Conceição, Departamento de Medicina Interna, Porto Alegre RS, Brazil.

**Keywords:** Bibliometrics, Emergencies, Neurology, Central Nervous System Diseases/Diagnosis and Therapy, Evidence-based Medicine, Critical Care/Standards, Bibliometria, Emergências, Neurologia, Doenças do Sistema Nervoso Central/Diagnóstico e Terapia, Medicina Baseada em Evidências, Cuidados Críticos/Normas

## Abstract

**Background** In 2012, the Neurocritical Care Society launched a compilation of protocols regarding the core issues that should be addressed within the first hours of neurological emergencies – the *Emergency neurological life support* (ENLS).

**Objective** We aim to evaluate this repercussion through a bibliometric analysis.

**Methods** We searched Scopus on October 2022 for articles mentioning ENLS. The following variables were obtained: number of citations; number of citations per year; number of publications per year; year of publication; research type; research subtype; country of corresponding author and its income category and world region; journal of publication and its 5-year impact factor (IF); and section where ENLS appeared.

**Results** After applying eligibility criteria, we retrieved 421 articles, published from 2012 to 2022. The mean number of citations per article was 17.46 (95% Confidence Interval (CI) = 8.20–26.72), while the mean number of citations per year per article was 4.05 (95% CI = 2.50–5.61). The mean destiny journal 5-year IF was 5.141 (95% CI = 4.189–6.093). The majority of articles were secondary research (57.48%; *n* = 242/421) of which most were narrative reviews (71.90%; *n* = 174/242). High-Income countries were the most prominent (80.05%; *n* = 337/421 articles). There were no papers from low-income countries. There were no trials or systematic reviews from middle-income countries.

**Conclusion** Although still low, the number of publications mentioning ENLS is increasing. Articles were mainly published in journals of intensive care medicine, neurology, neurosurgery, and emergency medicine. Most articles were published by authors from high-income countries. The majority of papers were secondary research, with narrative review as the most frequent subtype.

## Resumo

**Antecedentes** Em 2012, a Neurocritical Care Society lançou uma compilação de protocolos sobre as questões centrais que devem ser abordadas nas primeiras horas de emergências neurológicas – *Emergency neurological life support* (ENLS).

**Objetivo** Avaliar a repercussão do ENLS por meio de uma análise bibliométrica.

**Métodos** A base de dados Scopus foi utilizada em outubro de 2022 para a busca por artigos mencionando o ENLS. As seguintes variáveis foram obtidas: número de citações; número de citações por ano; número de publicações por ano; ano de publicação; tipo de pesquisa; país do autor correspondente e sua categoria de renda; revista de publicação e seu fator de impacto de 5 anos (IF); e seção onde o ENLS apareceu.

**Resultados** Os 421 artigos incluídos foram publicados de 2012 a 2022. A média de citações por artigo foi de 17.46 (intervalo de confiança (IC) 95% = 8.20–26.72), enquanto a de citações por ano por artigo foi de 4.05 (IC95% = 2.50–5.61). O IF médio por revista foi de 5.14 (IC95% = 4.19–6.09). A maioria dos artigos era de pesquisa secundária (57.48%; *n* = 242/421), dos quais a maioria eram revisões narrativas (71.90%; *n* = 174/242). Os países de alta renda foram os mais prolíficos (80.05%; *n* = 337/421 artigos). Não houve publicações de países de baixa ou média renda.

**Conclusão** Embora ainda baixo, o número de publicações mencionando o ENLS vem aumentando recentemente. A maioria dos artigos foram publicados em revistas de medicina intensiva, neurologia, neurocirurgia e medicina de emergência. Artigos de pesquisa secundária foram os mais comuns, com revisões narrativas sendo o subtipo mais frequente.

## INTRODUCTION

In 2012, the first edition of *Emergency neurological life support* (ENLS) protocols was published in *Neurocritical Care* , the official journal of the Neurocritical Care Society. 1 They were launched as a compilation of the core issues that should be addressed within the first hours of neurological emergencies, as recommended by experts and available evidence. Analogous to the Advanced Cardiovascular Life Support (ACLS) and Advanced Trauma Life Support (ATLS), the ENLS aims to standardize the initial approach to neurological emergencies. 2 3

Bibliometric analysis is a way to quantify research output and impact and has attracted the attention of the scientific community in recent years. 4 5 6 7 There is no consensus on how to perform this kind of study, where a high number of different variables may be assessed, depending on the purpose. For instance, relying on the number of articles, citations, and derived metrics allows for the quantification of the most prominent journals, countries, and research types in a specific field. Recently, several bibliometric analyses have been published in the fields of neurology, 8 9 neurosurgery, 10 11 and neurocritical care. 12

Unfortunately, the ENLS is relatively recent and is not as widespread as the ACLS and ATLS. However, it is mentioned by several recent guidelines within neurocritical care research, 13 14 15 demonstrating interest in standardized and systematic approaches to critically ill neurological patients. We are unaware of studies that assessed the repercussions of the ENLS in the scientific literature. Therefore, we aim to evaluate such impact through a bibliometric analysis.

## METHODS

### Search strategy

We searched *Elsevier's Scopus* on October 19 ^th^ , 2022 for articles mentioning ENLS in any part of the manuscript (title, abstract, keywords, text, or references). The exact search string is shown in Supplementary Material ( https://www.arquivosdeneuropsiquiatria.org/wp-content/uploads/2023/10/ANP-2023.0104-Supplementary-Material.docx ). Documents were excluded if they did not mention ENLS in any of these parts. Non-English studies, book chapters, conference abstracts, symposiums, discussion panels, erratum articles, as well as articles that could not have their full texts retrieved were also excluded.

### Bibliometric analysis

The full text of all included articles was analyzed. The following variables were obtained from each article: number of citations; number of citations per year; number of publications per year; year of publication; research type; research subtype; country of corresponding author and its income category and world region; journal of publication and its 5-year impact factor (IF); and local of appearance of ENLS (title, abstract, keywords, text, or references). The number of citations per year was not retrieved from articles published in 2022. Research types were classified as Primary, Secondary, Case Report, Animal/In vitro/Simulations, or Editorial/Comment. Research subtypes of primary research were further categorized as Interventional or Observational, while the subtypes of secondary research were subdivided into Narrative Review, Guideline/Consensus/Recommendations, and Systematic Review. The income category of countries and definition of world regions were retrieved from a Web site of *World Bank Country and Lending Groups* classifications ( https://datahelpdesk.worldbank.org/knowledgebase/articles/906519-world-bank-country-and-lending-groups ) in October 2022. The 5-year IF was obtained from the *Journal Citation Reports* Web site ( https://jcr.clarivate.com/jcr/home ) in November 2022. Besides the analysis of all articles, we also performed a separate analysis of articles that were ENLS protocols.

### Statistical analysis

We used SPSS version 23.0 for *Windows* for statistical analysis. We used *Prism Graphpad* for creating charts. For continuous variables, we obtained the mean, 95% confidence interval (95%CI), and percentiles 25th, 50th, and 75th. Continuous variables presented an asymmetrical distribution ( *p* < 0.001 on Kolmogorov-Smirnov test). Correlations were determined using *Spearman* rank correlation. We used the *Mann-Whitney* and *Kruskal-Wallis* tests for comparison of two means and three or more means, respectively. Pairwise *post-hoc* comparisons were performed after *Kruskal-Wallis* test. The level of significance was set at 5%.

## RESULTS

### General results

**Figure 1:**
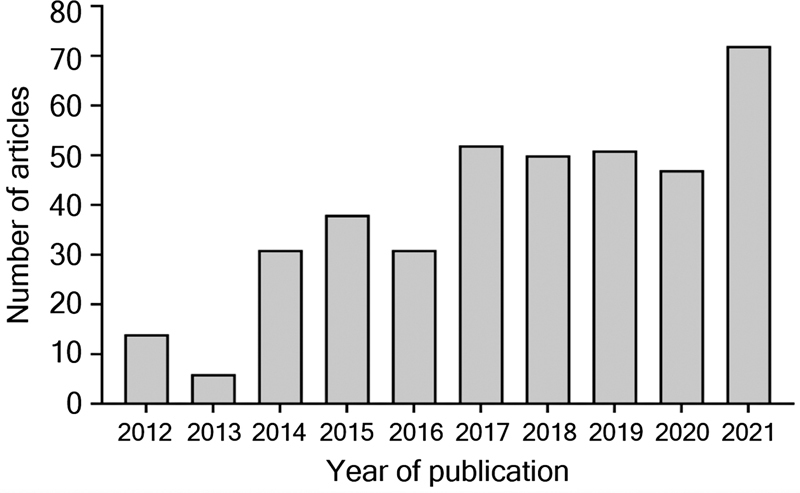
Longitudinal pattern of publications mentioning *Emergency Neurological Life Support* from 2012 to 2021.

After applying eligibility criteria to the 618 retrieved documents, a total of 429 articles were included. Of these, we could not obtain the full texts of 8 articles. The remaining 421 documents had their full texts retrieved and analyzed. The mean number of citations per article was 17.46 (95% CI = 8.20–26.72; 25th percentile: 1; 50th percentile: 5; 75th percentile: 15), while the mean number of citations per year per article was 4.05 (95% CI = 2.50–5.61; 25th percentile: 0.57; 50th percentile: 1.61; 75th percentile: 3.80). The ten most cited articles are detailed in Table 1 . There is an increasing number of publications mentioning ENLS over the years, as shown in Figure 1 .

**Table 1:** The ten articles mentioning the *Emergency neurological life support* with the highest number of citations

Title	Journal	No of citations	Country	Year
Guidelines for the Management of Spontaneous Intracerebral Hemorrhage: A Guideline for Healthcare Professionals from the American Heart Association/American Stroke Association	Stroke	1833	USA	2015
The European guideline on management of major bleeding and coagulopathy following trauma: Fourth edition	Critical Care	638	Switzerland	2016
Traumatic spinal cord injury: An overview of pathophysiology, models and acute injury mechanisms	Frontiers in Neurology	332	Canada	2019
Association between hyperoxia and mortality after stroke: A multicenter cohort study	Critical Care Medicine	169	USA	2014
Significance of arterial hyperoxia and relationship with case fatality in traumatic brain injury: A multicentre cohort study	Journal of Neurology, Neurosurgery and Psychiatry	95	USA	2014
Prehospital use of cervical collars in trauma patients: A critical review	Journal of Neurotrauma	93	Norway	2014
Intracerebral hemorrhage: An update on diagnosis and treatment	Expert Review of Neurotherapeutics	92	UK	2019
The critical care management of poor-grade subarachnoid hemorrhage	Critical Care	84	Canada	2016
High-dose midazolam infusion for refractory status epilepticus	Neurology	74	USA	2014
The critical care management of spontaneous intracranial hemorrhage: A contemporary review	Critical Care	74	Canada	2016

### Journal and 5-year impact factor

**Figure 2:**
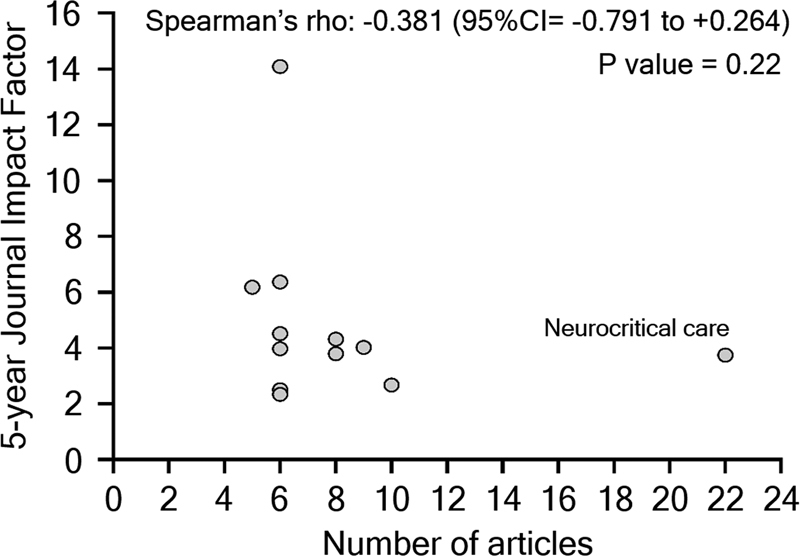
Correlation between the number of articles mentioning *Emergency Neurological Life Support* (ENLS) with the 5-year Impact Factors of destiny journals. Each small circle indicates a separate journal. Only journals with five or more articles were considered. ENLS protocols were excluded from the analysis.

Articles were published in 204 different journals, with a mean IF of 5.141 (95%CI = 4.189–6.093; 25th percentile: 2.879; 50th percentile: 3.748; 75th percentile: 4.764). The most prolific journal was *Neurocritical Care* (15.91%; *N* = 67/421) ( Table 2 ). Of these, 45 were official ENLS publications (protocols, descriptions of the ENLS, or descriptions of its updates). Considering only other publications (22 articles), *Neurocritical Care* remains the most prolific. A total of 49 journals did not have a 5-year IF. There was no correlation between the number of articles and 5-year IF ( Figure 2 ).

**Table 2:** Articles, citations, citations per article, and 5-year destiny journal impact factor analysis of publications mentioning the *Emergency neurological life support* stratified by journals. *N* = 421

Journal	Articles (% of total)	Citations per article, mean	Citations per year per article, mean	Destiny journal 5-year IF, mean
Neurocritical Care	67 (15.91)	3.74	2.33	3.75
Emergency Medicine Clinics of North America	10 (2.38)	2.67	3.03	2.68
Seminars in Neurology	9 (2.14)	4.02	1.18	4.02
Current Treatment Options in Neurology	8 (1.90)	3.79	2.07	3.80
Frontiers in Neurology	8 (1.90)	4.32	16.88	4.32
CONTINUUM Lifelong Learning in Neurology	7 (1.66)	NA	4.98	NA
Critical Care	6 (1.43)	14.08	30.47	14.08
Current Opinion in Critical Care	6 (1.43)	3.97	4.35	3.98
Journal of Stroke and Cerebrovascular Diseases	6 (1.43)	2.50	1.69	2.50
Neurologic Clinics	6 (1.43)	4.51	2.80	4.51
Resuscitation	6 (1.43)	6.35	1.47	6.36
World Neurosurgery	6 (1.43)	2.33	1.41	2.33
Journal of Neurology	5 (1.19)	6.17	3.11	6.17
Scientific Reports	4 (0.95)	5.51	2.66	5.51
Critical Care Clinics	4 (0.95)	4.76	4.22	4.76

Abbreviations: ENLS, Emergency Neurological Life Support. IF, Impact Factor.

Note: *Only citations up to 2021 were computed ( *N* = 392 articles).

### Country, region, and income category

**Figure 3:**
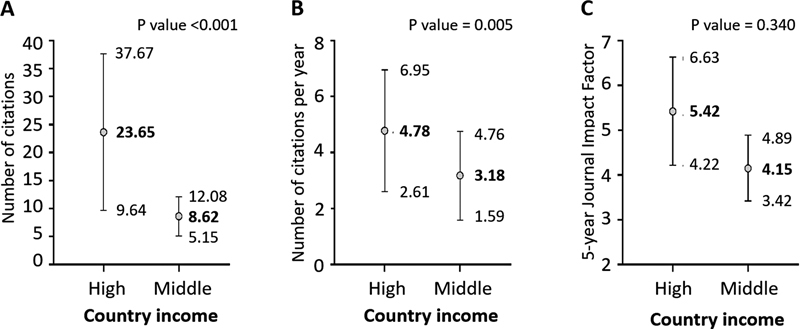
(A) Mean number of citations per article according to country income of articles mentioning *Emergency Neurological Life Support* (ENLS). Only citations up to 2021 were computed ( *N* = 392 articles). (B) Mean number of citations per year per article according to country income of articles mentioning ENLS. Only citations up to 2021 were computed ( *N* = 392 articles). (C) Mean 5-year destiny journal impact factor according to country income of articles mentioning ENLS. Data in A and B utilize complete years up to 2021 for analysis; discrepancies in article counts may be due to additional reports retrieved up to the search date in 2022.

Table 3 presents the most prominent countries, regions, and income categories within ENLS research. Articles were published by authors from 41 different countries. The USA was, by far, the most prolific (53.92%; *N* = 227/421 articles). The region with the highest number of papers was North America (57.96%; *N* = 244/421 articles). High-income countries were the most prolific (80.05%; *N* = 337/421 articles). There were no papers from low-income countries. Articles published by high-income countries had a higher mean number of citations and a higher mean number of citations per year than those published by middle-income countries ( Figures 3A and 3B ). Conversely, there was no difference in the mean 5-year IF between income categories ( Figure 3C ). All of the ten most cited papers were published by authors from high-income countries.

**Table 3:** Articles, citations, citations per year, and 5-year destiny journal impact factor analysis of publications mentioning the *Emergency neurological life support* (ENLS) research stratified by 15 countries with highest number of publications in the field, by world region and by country income. *N* = 421

Variable		Articles (% of total)	Citations per article, mean	Citations per year per article*, mean	Destiny journal 5-year IF, mean
**Country**	USA	227 (53.92)	20.11	3.94	5.46
	China	34 (8.08)	6.67	2.31	4.08
	Italy	19 (4.51)	7.94	2.82	3.88
	Canada	17 (4.04)	45.07	12.30	7.32
	South Korea	13 (3.09)	11.62	3.69	3.56
	India	12 (2.85)	3.16	1.65	2.39
	Japan	12 (2.85)	6.41	1.66	4.04
	Brazil	9 (2.14)	14.56	5.64	3.90
	Germany	9 (2.14)	9.11	2.29	4.53
	Switzerland	7 (1.66)	102.14	17.64	7.46
	UK	7 (1.66)	19.57	6.51	3.73
	Australia	5 (1.19)	2.40	0.29	3.89
	Egypt	5 (1.19)	0.20	0.00	N/A
	France	4 (0.95)	3.75	2.25	5.17
	Poland	4 (0.95)	9.00	1.20	2.47
**World region**	East Asia & Pacific	69 (16.39)	6.89	2.24	3.95
	Europe & Central Asia	67 (15.91)	20.00	4.84	4.39
	Latin America & Caribbean	15 (3.56)	11.46	4.66	4.27
	Middle East & North Africa	7 (1.66)	7.14	4.40	8.76
	North America	244 (57.96)	21.52	4.53	5.59
	South Asia	17 (4.04)	3.05	1.32	2.35
	Sub-Saharan	2 (0.47)	4.50	0.90	2.80
**Country income**	High income	337 (80.05)	20.25	4.40	5.21
	Upper middle income	56 (13.30)	7.55	2.95	4.11
	Lower middle income	28 (6.65)	3.64	1.77	3.19

Abbreviations: ENLS, Emergency Neurological Life Support. IF, Impact Factor; N/A, Not applicable.

Note: *Only citations up to 2021 were computed ( *N* = 392 articles).

### Research type and subtype

The research types and subtypes of papers mentioning ENLS are detailed in Table 4 . The most frequent research type was secondary research (57.48%; *N* = 242/421 articles), followed by primary research (30.64%; *N* = 129/421 articles). Among primary research articles, the research subtype with the greatest number of articles was Observational (92.25%; *N* = 119/129). Among secondary research, in turn, the most common research subtype was Narrative Review (71.90%; *N* = 174/242). Primary and secondary research were the study types of three and seven articles, respectively, of the ten most cited. There were no trials or systematic reviews from middle-income countries.

**Table 4:** Articles, citations, citations per article, and 5-year destiny journal impact factor analysis of publications mentioning the *Emergency neurological life suppor* *t* (ENLS) stratified by research type and subtype. *N* = 421

Research type/subtype	Articles (% of total)	Citations per article, mean	Citations per year per article*, mean	Destiny journal 5-year IF, mean
**Animal/in vitro/simulations**	16 (3.80)	1.83	6.37	3.84
**Case report**	17 (4.04)	0.57	0.47	1.36
**Editorial**	17 (4.04)	1.48	5.47	13.36
**Primary**	129 (30.64)	2.38	9.42	3.95
Interventional	10 (2.38)	2.13	8.80	4.00
Observational	119 (28.27)	2.40	9.47	3.94
**Secondary**	242 (57.48)	5.51	24.28	5.19
Guideline/consensus/recommendation	53 (12.59)	9.32	60.28	4.15
Narrative review	174 (41.33)	4.35	14.72	5.42
Systematic review	15 (3.56)	4.50	11.66	6.64

Abbreviations: ENLS, Emergency Neurological Life Support. IF, Impact Factor.

Note: *Only citations up to 2021 were computed ( *N* = 392 articles).

### ENLS occurrences and ENLS protocols

**Figure 4:**
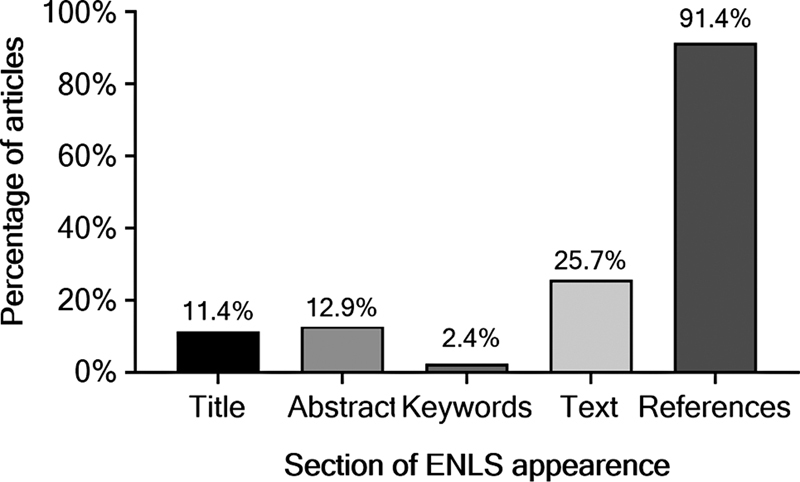
Section of appearance of *Emergency Neurological Life Support* in the included articles.

ENLS appeared more often in the References section, followed by the Text section ( Figure 4 ). A total of 10.69% ( *N* = 45/421 articles) were ENLS official publications. Of them, 41 were ENLS protocols and 4 were descriptions of the ENLS or descriptions of its updates. The ENLS Protocols were published by *Neurocritical Care* journal, receiving a mean number of citations of 13.13 (95% CI = 10.38–15.89; 25th percentile: 6.50; 50th percentile: 9.00; 75th percentile: 19.00) and a mean number of citations per year of 1.95 (95% CI = 1.53–2.37; 25th percentile: 0.93; 50th percentile: 1.60; 75th percentile: 2.73). Except for *Pharmacotherapy* - which had two published versions (2015 and 2017) -, all protocols had three published versions (2012, 2015, and 2017). In the first two editions, there was a “Traumatic Brain Injury” protocol. The third version, in turn, was named “Severe Traumatic Brain Injury.” The protocol with the highest mean number of citations among all versions was Intracranial Hypertension and Herniation (32.67 citations), followed by Intracerebral Hemorrhage (22.33 citations) ( Table 5 ). A total of 73.87% ( *N* = 311/421) articles only mentioned ENLS in the References section. When excluding ENLS official publications, only 65 articles (17.29%) mentioned ENLS in sections other than references.

**Table 5:** *Emergency neurological life support* modules and their mean number of citations per article and mean number of citations per year per article

ENLS module	Mean number of citations per article	Mean number of citations per year per article
Intracranial hypertension and herniation	32.67	4.61
2012	29	2.90
2015	50	7.14
2017	19	3.80
**Intracerebral hemorrhage**	**22.33**	**3.11**
2012	32	3.20
2015	15	2.14
2017	20	4.00
**Airway, ventilation, and sedation**	**19.67**	**2.82**
2012	26	2.60
2015	13	1.85
2017	20	4.00
**Status epilepticus**	**18.67**	**2.82**
2012	16	1.60
2015	20	2.85
2017	20	4.00
Traumatic brain injury*	**16**	**2.38**
2012	20	2.00
2015	8	1.14
2017	20	4.00
**Traumatic spine injury**	**14.67**	**2.23**
2012	16	1.60
2015	9	1.28
2017	19	3.80
**Resuscitation following cardiac arrest**	**13.67**	**1.85**
2012	18	1.80
2015	15	2.14
2017	8	1.60
**Pharmacotherapy**	**12.00**	**1.97**
2015	15	2.14
2017	9	1.80
**General**	**9.25**	**2.00**
2012	17	1.70
2015	7	1.00
2017	4	0.80
2020	9	4.50
**Meningitis and encephalitis**	**8.67**	**1.25**
2012	10	1.00
2015	8	1.14
2017	8	1.60
**Subarachnoid hemorrhage**	7.67	1.17
2012	7	0.70
2015	7	1.00
2017	9	1.80
**Approach to the patient with coma**	**7.00**	**0.85**
2012	13	1.30
2015	6	0.86
2017	2	0.40
**Acute ischemic stroke**	**5.67**	**0.82**
2012	6	0.60
2015	6	0.86
2017	5	1.00
**Acute non-traumatic weakness**	**5.67**	**0.84**
2012	6	0.60
2015	5	0.71
2017	6	1.20
**Spinal cord compression**	**4.33**	**0.56**
2012	8	0.80
2015	2	0.28
2017	3	0.60

Abbreviations: ENLS, emergency neurological life support.

## DISCUSSION

In this bibliometric study, we considered that the number of publications mentioning ENLS is low, with the majority of articles mentioning it only in the references section. However, this repercussion is increasing, demonstrating a growing interest in this subject ( Figure 1 ). Articles were mainly published in journals of intensive care medicine, neurology, neurosurgery, and emergency medicine. The majority of articles - including the most prominent - were published by authors from high-income countries. The majority of papers were secondary research, with narrative review as the most frequent subtype.

*Neurocritical Care* is the official journal of the *Neurocritical Care Society* , which is responsible for publishing ENLS protocols and other ENLS official publications. 1 Our study demonstrated that this was the journal with the greatest number of articles mentioning ENLS ( Table 2 ), even when not considering ENLS official publications. In addition, we did not find a correlation between destiny journal 5-year IF and the number of publications ( Figure 2 ). In fact, journals related to a specific field tend to have a lower IF than general medicine journals. 16 It does not mean, however, that these journals are less important. Actually, very specific journals - such as *Neurocritical Care* - attract more frequently the attention of a relatively small number of readers (i.e., subspecialists) in comparison with more general journals. Also, IF varies widely across specialties, precluding generalized comparisons. For example, while the greatest IF of a neurosurgical journal is 5.526, a total of 17 cardiology journals and 31 oncology journals have an IF greater than 10 (data retrieved from https://jcr.clarivate.com/jcr/home ).

In our study, high-income countries were the most prolific and the USA was the most prominent country ( Table 3 ). In addition, studies from high-income countries had a higher mean number of citations per article and a higher mean number of citations per year per article than studies from middle-income countries ( Figures 3A and 3B ). Several prior bibliometric analyses of neurology and neurosurgery topics also showed that the USA was, by far, the most prominent country. 9 10 12 The majority of papers mentioning ENLS were secondary research and a great portion were narrative reviews, which have a shallow level of evidence. Among primary research papers, we identified only 10 interventional - all of them from high-income countries ( Table 4 ). Actually, conducting original research in neurocritical care is challenging, especially interventional studies. It requires specialized training as well as specific resources to provide high-quality care, which are often scarce in developing countries. 17 18

The “first hour” of neurological emergencies is critical, since a fast workup may significantly improve patients' outcomes. For instance, the recognition of ventilatory distress in patients with acute non-traumatic weakness may lead to timely intubation. 19 Similarly, the detection of early signs of intracranial hypertension in a patient with severe traumatic brain injury would possibly prevent brain herniation. 20 By recognizing the importance of this fast workup, health professionals and institutions would probably increase their interest in ENLS certification and training. In the current study, “Intracranial Hypertension and Herniation” ( Table 5 ) was the ENLS module with the greatest impact. This is one of the most basic and general modules, since various brain pathologies may lead ultimately to intracranial hypertension and brain herniation.

Even with the overt importance of a proper systematic and concise approach for neurologic emergencies, the demand for certification on ENLS is still limited when compared with its analogs in trauma and advanced cardiovascular support (ATLS and ACLS, respectively). This fact may find an explanation in the time of activity of each program. The ATLS was introduced in 1980 3 and has now been taught to more than 1 million physicians in more than 80 countries worldwide (data retrieved from facs.org/quality-programs/trauma/education/advanced-trauma-life-support/ in December 2022). 3 Likewise, the ACLS was presented in the middle 70s as the result of the second National Conference held on cardiopulmonary resuscitation (CPR) and emergency cardiac care (ECC) in 1973, which recommended delivery of advanced cardiac life support by trained personnel be required of all life-support units and hospitals on an integrated, community-wide basis and that training in CPR and techniques of ECC be under standards set by the AHA. 21 22 Every year, the ACLS course is cumulatively accessed throughout the world by over 1.3 million candidates. 22 As ENLS consolidates over the following years in different localities, advocacy efforts and community engagement seem to be the most promising way to foretaste acute neurological care, as done by ATLS and ACLS.

The accessibility of ENLS protocols may indeed influence their adoption and utilization in the scientific and medical communities. The protocols, devised by the Neurocritical Care Society, are not widely and freely available but rather necessitate a purchase. This financial barrier could potentially limit the dissemination and implementation of ENLS, particularly in low- and middle-income countries or among individual practitioners and smaller healthcare establishments with restricted budget allocations for accessing scientific materials. It is plausible that this financial aspect could subsequently influence the number of research articles discussing or utilizing ENLS, as the researchers might prefer or be constrained to utilize freely accessible guidelines and protocols. Thus, while ENLS provides a structured and standardized approach toward managing neurological emergencies, its broader impact on clinical practices and research might be restrained by its limited accessibility. Future initiatives or collaborations that facilitate wider, cost-effective access to these protocols might enhance their reach and implementation globally, fostering further research and potentially elevating patient care standards in neurological emergencies.

In the authors' opinion, the repercussions of ENLS on scientific literature are still low. The majority of papers only mentioned ENLS in the references section ( Figure 4 ). When excluding ENLS official publications, only 65 articles mentioned ENLS in the title, abstract, keywords, or text. The ENLS protocols are being updated constantly and the number of certified professionals is increasing. However, only 34 institutions in the USA and 9 in other countries require ENLS certification for some or all of their students and/or care providers (data retrieved from https://enls.neurocriticalcare.org/about/enls-institutions in December 2022). In addition, live ENLS courses have been held in 35 countries (data retrieved from https://enls.neurocriticalcare.org/about/statistics in December 2022). Therefore, there is still much to be done to ensure ENLS dissemination and that more institutions recognize the importance of this certification.

## Limitations

The present bibliometric study has some limitations. First, only one database ( *Elsevier's Scopus* ) was used. However, this is one of the most comprehensive databases that track citation patterns, including over 71.2 million records post-1969 with reference. 23 The decision to utilize Scopus exclusively was also influenced by its comprehensive inclusion of databases such as Medline and Embase, as well as its provision of reliable citation count data, crucial for executing robust bibliometric analyses. It's noteworthy that other databases like Lilacs and Scielo, while being significant, do not offer citation count data, and Google Scholar, which encompasses a wide range of internet references, was not chosen to ensure the impact assessment remained strictly within the bounds of scientific literature. Second, we considered only the country and its respective region and income category based on the corresponding author's affiliation. These affiliations may differ from other authors who also contributed to the studies. Third, recent articles did not have the same time exposure when compared with older articles, certainly affecting the total number of citations. Therefore, we also analyzed the number of citations per year per article, which helps to minimize these discrepancies. Fourth, only English-language articles were included. Articles published in other languages would possibly impact our findings. Fifth, we evaluated the repercussions of ENLS only on the scientific literature.

In conclusion, we found that the ENLS repercussion on the scientific literature is low, although increasing. The majority of papers that mentioned ENLS were secondary research and were mainly published by authors from high-income countries. Most articles only mentioned ENLS in the references section. Usually, the health professionals responsible for the initial management of neurocritically ill patients are not specialists in neurocritical care, who are frequently contacted after the first measures. Therefore, not only neurocritical care specialists but also all professionals who are likely to be exposed to scenarios of neurological emergencies should receive ENLS training. To increase the ENLS repercussion and training, some measures might be useful. We suggest making ENLS training a requirement for emergency department and intensive care professionals; making ENLS available in several languages, and adding regional particularities to the protocols with partnerships with local medical societies.
